# Malignant Rhabdoid Tumor of the Kidney in a Child With Xeroderma Pigmentosum: Incidence or Coincidence?

**DOI:** 10.7759/cureus.27049

**Published:** 2022-07-20

**Authors:** Abdelilah Lahmar, Ghanam Ayad, Hiba Ramdani, Othman Moueqqit, Imane Kamaoui, Miry Nadir, Amal Bennani, Noufissa Benajiba

**Affiliations:** 1 Family Medicine, Mohammed VI University Hospital, Oujda, MAR; 2 Pediatrics, Mohammed VI University Hospital, Oujda, MAR; 3 Radiology, Mohammed First University of Oujda, Oujda, MAR; 4 Pathology, Mohammed First University of Oujda, Oujda, MAR; 5 Pediatrics, Mohammed First University of Oujda, Oujda, MAR

**Keywords:** kidney, abdominal mass, case report, rhabdoid tumor, xeroderma pigmentosum

## Abstract

Malignant rhabdoid tumor of the kidney (MRTK) is a rare aggressive malignant rhabdoid tumor that mainly affects children. At the onset of the disease, the usual clinical manifestations are gross hematuria, abdominal pain, and abdominal distension. The prognosis remains poor. Patients with rhabdoid tumors (RT) are treated according to institutional preferences that combine surgery, radiation therapy, and chemotherapy. The authors present the rare case of a child with xeroderma pigmentosum (XP) who presented with an abdominal mass accompanied by hematuria and abdominal pain. The radiological and histological results were congruent with the MRTK. The patient received preoperative chemotherapy but unfortunately died of septic shock. This case highlights the importance of being aware of MRTK and its fatal complications, as well as the increased risk of kidney tumors in patients with XP.

## Introduction

Rhabdoid tumors (RT) are rare and more aggressive malignancies that predominantly occur in infants and young children [1]. Depending on the anatomical localization, the 3 primary localizations of RT are atypical teratoid/rhabdoid tumor (AT/RT), extra-renal non-cranial rhabdoid tumor, and malignant rhabdoid tumor of the kidney (MRTC) [2]. Due to its rarity in clinical practice, epidemiological studies are limited [3].

The most frequently reported symptoms are abdominal masses and hematuria which can often be detected in the metastatic stage. The definitive diagnosis is made by detecting the SWItch/sucrose nonfermentable (SWI / SNF) mutation-related matrix-associated actin-dependent regulator of chromatin subfamily B1 (SMARCB1) gene or by negative immunohistochemistry integrase interactor 1 (INI-1) [4]. Surgery, postoperative radiotherapy, and chemotherapy are the main approaches to treating patients with MRIK. So far, however, there is no uniform standard for its treatment [5]. The prognosis is poor [6].

Xeroderma pigmentosum (XP) is a rare autosomal recessive disease characterized by increased sensitivity to the harmful effects of ultraviolet light due to a lack of DNA nucleotide excision repair (NER) with a high incidence of skin cancer [7]. In addition, some malignant renal tumors such as nephroblastoma, malignant mixed epithelial, and stromal tumors of the kidney have been reported [5]. No case has been published reporting the association between xeroderma pigmentosum and malignant rhabdoid tumor of the kidney.

In light of this, we present the case of a seven-year-old female patient with xeroderma pigmentosum followed since the age of six months, and had a malignant rhabdoid tumor of the kidney with pulmonary metastases, treated with chemotherapy. The relationship between xeroderma pugmentosum and the malignant rhabdoid tumor is discussed. 

## Case presentation

**Figure 1:**
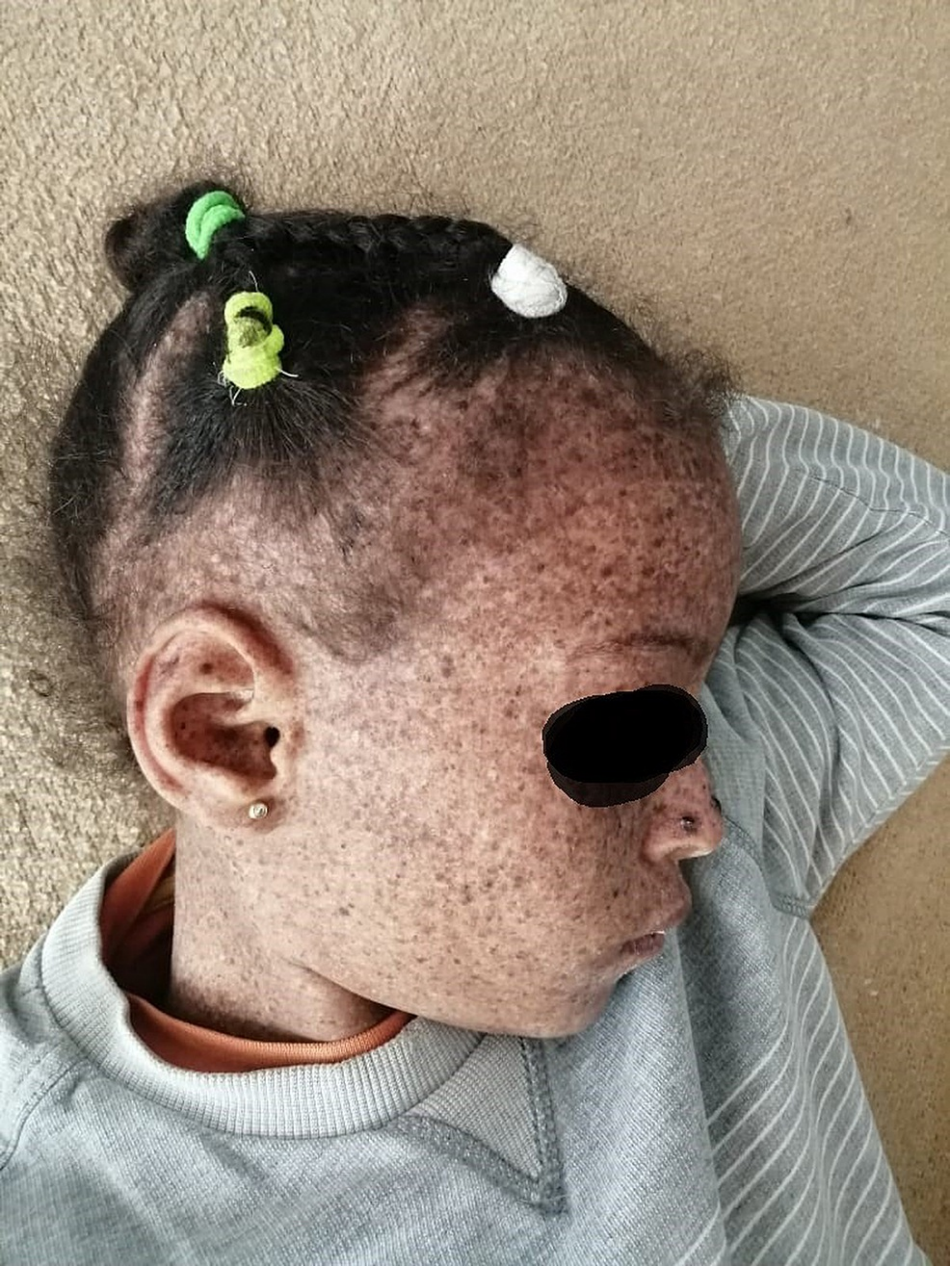
The clinical appearance of xeroderma pigmentosum covering the face of the child

A seven-year-old consanguineous girl had been followed by a dermatologist since the age of six months for her xeroderma pigmentosum (Figure 1), with no other specific personal or family history noted. She is the eldest of her two healthy siblings. The patient presented with a week-long chief complaint of abdominal pain associated with fever and deterioration in general condition with no associated urinary signs. The parents mention that the pain started three months ago and was intermittent. The patient presented with a chief complaint of abdominal pain for one week associated with fever and deterioration of general condition without accompanying urinary signs. The parents mention that the pain started three months ago which was intermittent. 

On physical examination, the patient is conscious and alert with altered general condition. Her weight of 20 kg, height of 125 cm, heart rate of 92, respiratory rate of 32, blood pressure of 94/62 with a temperature of 38 C. The urine dipstick shows hematuria without leukocyturia. Dermatological examination shows diffuse hyperpigmentation skin lesions predominantly in the face and trunk. Abdominal examination is normal. Renal system examination showed a positive bimanual palpation. Examination of the lymph node areas found a left submandibular lymphadenopathy less than 1,5 cm, painless, hard and without inflammatory signs. Other systems were grossly intact.

Abdominal ultrasound showed an enlarged right kidney containing a polar mass of oval appearance measuring 45 mm x 36 mm, well defined, regular contours, and heterogeneous echogenicity. The contralateral kidney was without abnormalities. 

**Figure 2:**
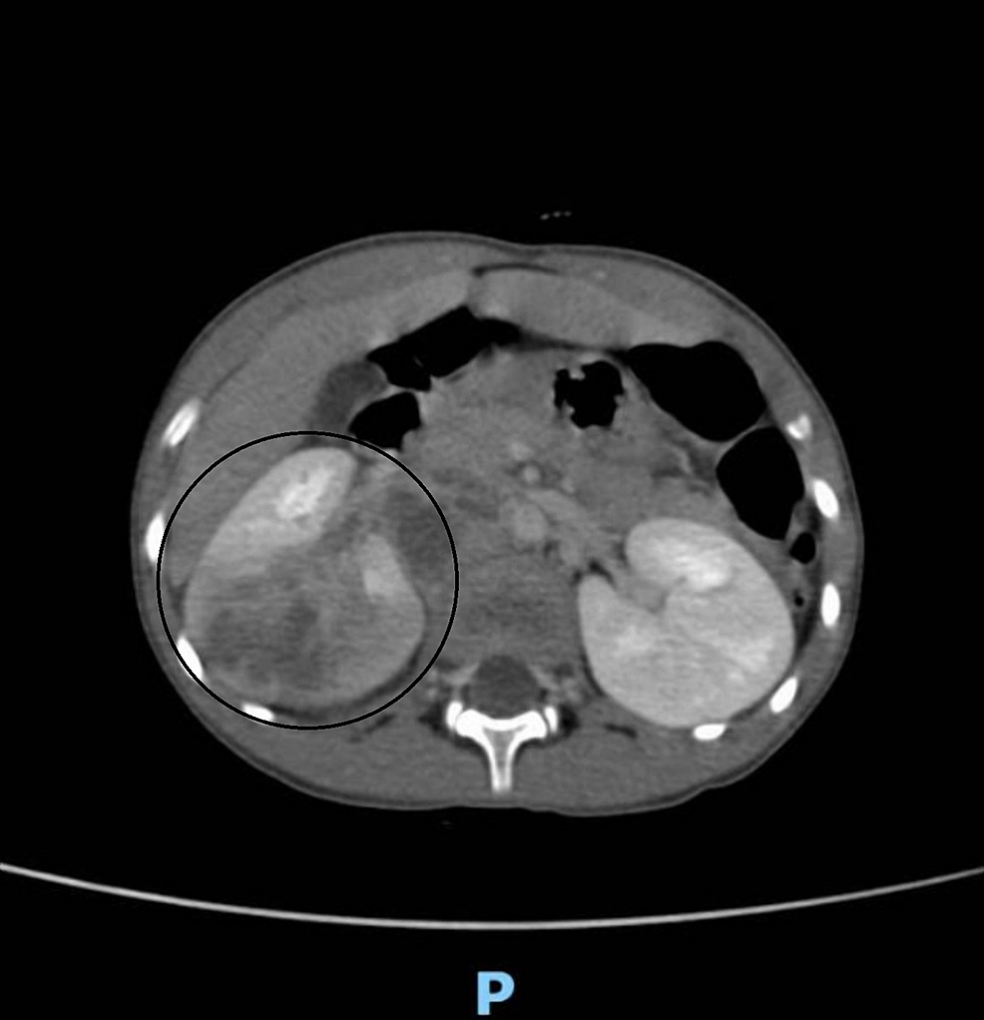
Transverse view of the patient’s abdominal computed tomography revealing an upper right polar renal tumor process with isodense contours, enhancing heterogeneously after injection of contrast agent

In order to better characterize this kidney mass, a thoraco-abdomnino-pelvic CT was performed. It showed the presence of an upper right polar renal tumor process measuring 58 x 51 x 53 mm with poorly defined, isodense contours, enhancing heterogeneously after injection of contrast agent, containing areas of necrosis (Figure 2). Invading segment VI of the liver, coming into contact with the right poster lateral abdominal wall associated with retroperitoneal lymphadenopathy. The test has also shown the presence of a pulmonary parenchymal nodule of suspicious appearance in the upper right lobe.

Laboratory tests show red cells blood 3.03. 10^6 ^million/mm^3 ^(normal range, 4,5-5,1 million/mm^3^), hemoglobin 8,6 g/dL (normal range, 11,2-16,5 g/dL), platelet count of 585,000/μl (normal range, 140-400/µl), international normalized ratio (INR) of 1,42, activated partial thromboplastin time (aPTT) of 2,25, Uric acid 1.5 mg/dL (normal range, 2,0-7,0 mg/dL), calcium 9.5 mg/dL (normal range; 8.8-10.8 mg/dL), potassium 5.4 mEq/l (normal range, 3.5-5.5 mEq/l), sodium 133 mEq/l (normal range, 135-145 mEq/l), LDH 299 UI/L (normal range, 143-290 U/L), urea 15 mg/dL (normal range, 5-18 mg/dL) and creatinine- 3,18 mg/dL (normal range, 0.31-0.61 mg/dL). Liver function test was unremarkable.

The differential diagnoses at that time were: Wilms tumor, renal cell carcinoma, congenital mesoblastic nephroma, and rhabdomyosarcoma.

**Figure 3:**
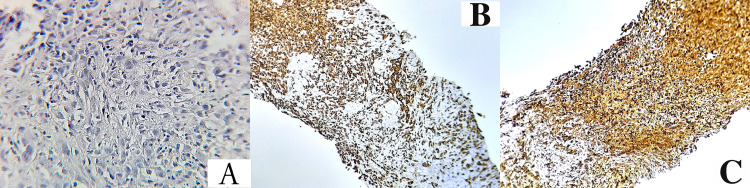
Histological examination of the tumor shows a diffuse proliferation of spindle cells arranged in diffuse sheets with a rhabdoid appearance, tumor cells shows evident atypia with enlarged, irregular and hyperchromatic nuclei, the cytoplasm is mostly eosinophilic (A). Several mitotic figures were seen. Immunohistochemical staining shows a positivity for cytokeratin (B), vimentin (C), and Cyclin-D1. Tumor cells were negative for WT1, CD34, PS100, Ck7, desmin, CD10 and LCA. LCA:  leukocyte-common antigen

A lymphadenopathy biopsy was performed. The histopathological examination revealed a tumor proliferation arranged in sheets made of spindle-shaped rhabdoid appearance with the presence of tumor necrosis. Immunohistochemistry demonstrated focal expression of cytokeratin and vimentin by tumor cells (Figure 3) with lacking INI-1 immunohistochemical staining.

Based on clinicopathological findings, the diagnosis of a malignant rhabdoid tumor of the kidney was made. According to the National Wilms Tumor Study (NWTS) staging system modified by The Children's Oncology Group (COG), the tumor is staged IV. The patient was put on preoperative chemotherapy according to the Groupe Franco Africain d'Oncologie Pédiatrique nephrology (GFAOP NEPHRO) protocol. Unfortunately, the patient died of septic shock.

## Discussion

The malignant rhabdoid tumor of the kidney (MRTK) is a highly aggressive tumor and was discovered in 1982 by Haas et al. The term rhabdoid is derived from the histological similarity of tumor cells with rhabdomyoblasts [8, 9]. Epidemiologically, the proportion of MRTK is estimated at 0.9% - 2% [8]. According to the German Children's Cancer Registry, the absolute number of reported cases of rhabdoid tumors between 2010 and 2015 was 139, with male prevalence in all cases, in which only 9% of the MRTK were mentioned [3]. It occurs more frequently in children aged 2-year-old or older and is low in the neonatal form [4].

Low birth weight, multiple births, premature births, and late-term delivery have been reported to be associated, but it has not been established whether these factors contribute to the onset of the disease [10].

The majority of rhabdoid tumors show a genomic alteration mainly in the tumor suppressor gene SMARCB1 and to a lesser extent in the other genes. Of the newly diagnosed patients, 25-30% have a germline change in SMARCB1 that predisposes them to cancer [11, 12]. Rhabdoid tumors develop primarily through the SMARCB1. The SWI/SNF complex plays a crucial role in the regulation of gene transcription and influences multiple signal transduction pathways involved in cancer [13].

In patients with XP, the pathophysiology of tumor development in internal organs that are not exposed to UV radiation is not understood [5]. The XPA gene is known to carry instructions for producing proteins that aid in the repair of damaged DNA. This gene encodes a number of proteins, including XPC (Xeroderma pigmentosum, complementation group C) and XPA (Xeroderma pigmentosum, complementation group A), which recognize nuclear damage and recruit the nucleotide excision repair (NER) pathway, and thus attract SWI / SNF to the damage site, which in turn remodels the nucleosome and promotes the whole excision nuclease as well as the final repair of damage. Thus, genetic defects in XP subtype with SWI / SNF mutation may promote the accumulation of mutations to take place due to the ineffective DNA repair system which promotes tumor development [14]. According to a meta-analysis, XP subtype C had a greater risk of bladder cancer due to a repair deficiency in bladder cell DNA exposed to tobacco [5]. In Table 1, we list the cases of renal tumors in patients with xeroderma pigmentosum that have been published in the literature, along with their features.

Clinically, the patients present with abdominal mass, hematuria, and abdominal pain. MRTK tends to metastasize very early. More than two-thirds of children with MRTK are in the advanced stages of the disease with distant metastases to the lungs and/or brain [8]. In a retrospective study of 14 cases of children with a malignant rhabdoid tumor of the kidney, the first symptoms of this group mainly include macroscopic hematuria and abdominal mass. In addition, 12 patients were found with pulmonary metastases, two patients with liver metastases, and four with lymphadenopathy metastases [15].

Laboratory tests can reveal anemia, microscopic hematuria, and hypercalcemia due to the tumor's ectopic production of parathyroid hormone-related protein [16].

MRTK cannot be distinguished from other renal malignancies by any pathognomonic imaging characteristic. However, some features may enhance suspicions about MRTK over others. More favorable for MRTK is the presence of a large, lobulated mass in the center or periphery of the kidney with areas of hemorrhage or necrosis. Calcifications occur frequently in MRTK and are often linear or curvilinear [17].

Histologically, the tumor cells are characterized by sheet trabeculae with eccentric vesicular nuclei and eosinophilic cytoplasmic inclusions. The most useful ultrastructural finding is the intermediate filaments in the cytoplasm which are in favor of rhabdoid inclusions. On immunohistochemical examination, tumor cells are positive for vimentin, epithelial membrane antigen, and/or cytokeratin, and the INI1 staining is lacking in malignant rhabdoid tumors [18].

To date, there is no standard treatment for patients with RT. Until recently, patients with RT were treated according to institutional preferences that combined surgery, radiation therapy, and chemotherapy. The EU-RHAB registry for RT of any anatomical origin recommends total resection, conventional chemotherapy (vincristine, dactinomycin, cyclophosphamide, doxorubicin, ifosfamide, carboplatin, etoposide), intrathecal methotrexate (MTX) and radiotherapy [19].

The tumor has an overall poor prognosis. Recent research found that only one patient out of 35 survived after a five-year follow-up [4]. The prognosis depends on several parameters, including age, comorbidities, metastases, early detection, and treatment. Interestingly, it has been found that the long-term effects of treatment include both medical ( cerebro- and cardiovascular, endocrine, neurological…) and non-medical complications (neurocognitive, psychological,..) can sometimes be life-threatening and impairs quality of life. In a study with 199 cases, 60% of the survivors of the rhabdoid tumor suffered from therapy-related long-term effects. Neurological deficits and endocrinopathy were more common, as were neurocognitive and psychosocial delays. Three cases of AML were found secondary to treatment [3].

**Table 1:** The characteristics of malignant renal tumors in patients with xeroderma pigmentosum found in the literature N/A : not applicable; CT Scan: computed tomography

	Age (years/sex)	Clinical presentation/physical examination	Radiological features	Histological diagnosis	Management	Prognosis
Lahlimi F et al. (2015) [20]	5-year-old boy	A mass in the left hypochondrium with lumbar tenderness and pigmented lesions diffused all over the body, without lymphadenopathy or organomegaly	Abdominal ultrasound: a left kidney mass measuring 78/86 mm, necrosed and multi-loculated without signs of extension. CT scan: 84/87 mm cystic left renal tumor with a focus left basal alveolar condensations and 2 left subpleural nodules	Nephroblastoma with anaplastic component	The GFAOP protocol (Franco-African pediatric oncology group) - nephroblastoma 2005, metastatic form	The child is in complete remission with a follow-up of 12 months
Visweswara RN et al. (1997) [21]	Case 1: 17-year-old female Case 2: 16-year-old female	Repeated attacks of ureteric colic for 18 months associated with irritative voiding symptoms. Diffuse pigmentation of the skin all over the body, palpable and non-ballotable mass in the left subcostal region. Progressive increasing left flank pain and irritative voiding symptoms for a period of 3 months. Café-au-lait pigmentation of the whole body, a well-defined, non-tender, firm-to-hard renal mass (15 x 12 x 10 cm) was palpated in the left flank	Abdominal ultrasound: well-defined cystic left renal mass CT scan: large necrotic tumour (15 x 15 cm) involving the lower portion of the left kidney, abutting against the anterior abdominal wall and indenting the descending colon CT scan: mass (15 x 10 cm) of mixed echogenicity replacing almost the entire left kidney and distorting the pelvicalyceal system	Wilms’ tumour with predominant mesenchymal elements Wilms’ tumor	Left radical nephrectomy with chemotherapy Radical left nephrectomy with multidrug chemotherapy	Intolerance to chemotherapy with deterioration in general health and death at home. Discontinuation of chemotherapy due to his intolerance. Hospitalization of the patient after 6 months by the appearance of pulmonary, bone and hepatic metastases and consequently her death
Smichi I et al. (2015) [22]	27-year-old female	Clinical picture of acute bowel obstruction. On physical examination, the abdomen was distended and bloated. The left lumbar region was tender	CT Scan: voluminous polylobed, multi-compartmentalized left renal mass measuring 18 cm, surrounded by a pseudo-capsule and enhancing heterogeneously after injection of the contrast, compressing the transverse colon against the abdominal wall causing colonic and ileal upstream dilatation	Mixed epithelial and stromal tumor of the kidney	Left radical nephrectomy	After the surgery, the patient was lost to follow-up, she did not reconsulted
Tomás M et al. (1989) [23]	N/A	N/A	N/A	N/A	N/A	N/A
Boulma R et al. (2021) [5]	14-year-old adolescent	Abdominal pain and total hematuria for 4 weeks. thoracoabdominal diffuse hyperpigmentation skin lesions with a firm non-tender palpable mass situated in the right lumbar and hypochondrium extending to the midline	Abdominal ultrasound: Right superior solido-cystic renal mass measuring 9 cm CT scan: the presence of an upper right polar renal mass, with a cystic and solid component, measuring 10 × 9 × 7 cm significantly enhanced after injection of contrast agent	High-grade leiomyosarcoma	Transperitoneal total ureteronephrectomy with an adjuvant chemotherapy	At 12 months follow-up, the patient is in total remission with no recurrence in the CT scan

## Conclusions

Malignant rhabdoid tumor of the kidney is a rare entity among all renal tumors. Diagnosis is based on clinical, radiological, and histological features. Despite advances in pediatric oncology, it has a poor prognosis, especially in the absence of a unified therapeutic regimen. Further studies are needed for broader aspect knowledge, including genetics, to develop a consensus treatment that effectively addresses the disease and improves patient prognosis.
